# Carcinome épidermoïde du sein: à propos d’un cas en Mauritanie

**DOI:** 10.11604/pamj.2019.33.143.18375

**Published:** 2019-06-25

**Authors:** Zein Ahmed, Ahmedou Moulaye Idriss, Ahmed Heiba, Isselmou Sidi

**Affiliations:** 1 Centre Hospitalier Cheikh Zayed de Nouakchott, Faculté de Médecine de Nouakchott, Mauritanie; 2 Centre Hospitalier National Nouakchott, Faculté de Médecine de Nouakchott, Mauritanie

**Keywords:** Carcinome, épidermoïde, sein, Cell, carcinoma, breast, lymph node dissection

## Abstract

Le carcinome épidermoïde primitif (CEP) ou carcinome de cellules squameuses du sein est une tumeur maligne rare des cancers mammaires. D'origine métaplasique, son histogenèse et son pronostic sont controversés ainsi que sa présentation clinique et mammographique n'ont pas de particularité par rapport aux autres cancers. Le CEP du sein se caractérise par une évolution rapide et un traitement non codifiée. Le but de ce travail est de rapporter les aspects cliniques et évolutifs d'un nouveau cas de CEP avec une revue de littérature.

## Abstract (en)

Primary epidermoid carcinoma (PEC) also known as squamous cell carcinoma of the breast is a rare tumor accounting for 0.1% to 2% of all breast cancers. It is a metaplastic carcinoma; its histogenesis and prognosis are controversial as well as its clinical and mammographic appearances which are not characteristic compared to other cancers. PEC is characterized by a rapid evolution and treatment is not codified. The purpose of this study is to report the clinical and evolutionary features of a new case of PEC and to conduct a literature review.

## Introduction

Le carcinome épidermoïde primitif (CEP) est une entité connue et réputée plus fréquente dans la peau et dans d´autres organes tapissés de cellules squameuses telles que l´œsophage et l'anus. Le CEP du sein est très rare, il représente 0.1% à 2% de tous les carcinomes du sein invasifs. En raison de sa rareté et de la confusion entourant sa définition, la CEP du sein est difficile à analyser. Bien que son aspect morphologique et ses variations histologiques soient identiques à ceux provenant d´autres tissus, son diagnostic n'est évoqué qu'après une biopsie. Peu de cas ont été signalés jusqu´à présent dans la littérature. Il s'agit d'une tumeur très agressive, avec récepteurs hormonaux négatifs et réfractaire au traitement, de mauvais pronostic. Le traitement chirurgical a les mêmes indications que les carcinomes canalaires. L'indication et le type d'un traitement adjuvant restent controversés du fait de la rareté de la tumeur. Le pronostic semble être comparable à celui des carcinomes indifférenciés. Nous rapportons un cas de cette malignité rare.

## Patient et observation

Madame S.Z est âgée de 70 ans mère de 2 enfants ayant comme antécédent, une hypertension artérielle suivie et traitée. Elle a présenté en décembre 2007, une masse nodulaire du sein gauche évoluant depuis deux mois au niveau du quadrant supéro externe (QSE) gauche de taille de 2 cm, cette masse est mobile sans adénopathie axillaire associée. L´échographie mammaire a montré une formation hétérogène de contours réguliers qui prend la taille d'une Amande. La mammographie a montré une opacité à contour régulier. L´échographie abdominale, la radio du poumon et le bilan biologique sont normaux. Un frottis cervico-vaginal demandé était normal. La cytopnction a montré des cellules cancéreuses. Une tumorectomie avec curage ganglionnaire axillaire gauche ont été pratiqués. L´étude histologique a mis en évidence un carcinome épidermoïde avec des marges de section saine et trois ganglions sur sept étaient envahis. Le diagnostic d´un cancer épidermoïde du sein gauche a été retenu et un traitement complémentaire a été démarré par une radiothérapie sur la paroi et sur les aires ganglionnaires. La chimiothérapie a été démarré (FAC), après 4 cures une aplasie médullaire développée par la patiente qui a nécessité une hospitalisation à la réanimation pendant 10 jours avec transfusion sanguine et la chimiothérapie a été arrêtée. L´évolution a été marquée par l´apparition d´un nodule après 12 mois au niveau de la cicatrice, puis une exérèse a été réalisée et a confirmé la récidive locale d´un carcinome épidermoïde. Une mastectomie a été pratiquée et l'examen histo pathologique n´a pas montré de résidu tumoral. Vue les antécédents de la patiente (aplasie médullaire lors de première chimiothérapie et l´âge de patiente 73 ans la décision a été de ne pas faire un traitement complémentaire. La patiente a fait un suivi régulier pas de récidive local ou métastase à distance avec un recul de 10 ans après le diagnostic.

## Discussion

Les carcinomes épidermoïdes primitifs du sein sont une entité rare et peu connue. Leurs fréquences sont de 0.1% à 2% de l´ensemble des carcinomes du sein. L'ethiopathogenie reste controversée. S'agit-il d'une entité à part entière? Ou d'un adénocarcinome qui subit une métaplasie épidermoïde partielle ou totale [1]. Selon l'OMS il fait partie des carcinomes canalaires infiltrant comportant des remaniements totaux ou partiels. On parle de carcinome épidermoïde pure quand il s'agit de métaplasie totale. L'origine est glandulaire, pour certains auteurs elle est myoépithéliale [2]. Le diagnostic histologique de ce type de cancer n'est établie qu'après avoir éliminé une origine cutanée ou mamelonnaire, une métastase mammaire d´un carcinome épidermoïde à distance et enfin une composante glandulaire significative au sein de la tumeur (Carcinome muco-épidermoïde). L'âge de survenu du carcinome épidermoïde du sein est similaire aux autres carcinomes mammaires il touche les femmes entre 31 et 83 ans avec un pic à 55 ans [3]. Aucun facteur prédisposant a été décrit, par contre la notion de traumatisme locale a été observé [4].

L'aspect clinique et radiologique de ces tumeurs n´est pas spécifique elle peut se manifester comme une masse de taille différente entre 2-6 cm parfois abcédée ou enkystée avec des ulcérations de la peau dans certain cas ce qui pose un problème de son origine primitive ou secondaire. La tumeur peut être bilatérale, mais aussi peut se manifester à gauche comme à droite. Sur le plan radiologique l'échographie peut montrer une masse d'aspect kystique. A la mammographie l'aspect n'est pas spécifique: arrondie, sans spécule. Pour certains auteurs la présence d´une masse kystique ou abcédée chez une femme âgée doit faire penser à un carcinome canalaire du sein. Le diagnostic peut se faire grâce à une aspiration cytologique ou mue biopsie. Notre patiente a bénéficié d´une cytologie qui a permis le diagnostic. L'examen histologique est indispensable, il a la même architecture et les mêmes caractères cytonucleaires retrouvés dans d'autres sites. Donc le diagnostic du cancer se fait après avoir éliminé une métastase d'un carcinome épidermoïde à distance ou une extension locale d´une atteinte cutanée ou mamelonnaire. L'immunohistochimie montre une expression de cellules tumorales épithéliales des chromeratines de haut poids moléculaire (CK14, CK6, 6 et CK17) [5]. La recherche des récepteurs tumoraux est souvent négative dans la plupart de cas. Par contre sheen *et al.* [6] ont rapporté un cas de cancer épidermoïde avec récepteurs hormonaux positifs. Dans notre cas les récepteurs étaient négatifs.

Le carcinome épidermoïde est peu lymphophile dans 70% des cas le curage est dépourvu de métastase ganglionnaire [7]. Pour notre cas le curage était positif 3 ganglions+/7ganglions. Les études moléculaires démontrent hyper expression de la protéine p53 et de ces marqueurs de l´angiogenèse VEGF et HIF -1alpha [8]. Le traitement est similaire à celui des cancers canalaires infiltrant de même taille et stade [9]. Le traitement est basé sur la mastectomie avec curage ganglionnaire axillaire pour les tumeurs de grande taille suivis d´une radiothérapie et d´une chimiothérapie. Le traitement conservateur est possible pour les tumeurs de petite taille. L'hormonothérapie n'a pas sa place, vue l'absence de l'hormonodependence de ce cancer. *Le traitement neoadjuvant* utilisé par certains auteurs comme Dejager *et al.* [4] a la base de cis- platine et 5 fluoracil a permis la réduction du volume tumorale avant de faire une chirurgie. Le pronostic du carcinome épidermoïde est sujet de controverse dans la littérature il a le même pronostic des cancers canalaires infiltrant du sein du même stade [10].

## Conclusion

Les carcinomes épidermoïdes du sein sont rares, ils n'ont pas de spécificité clinique ou radiologique. Le traitement se repose sur la chirurgie la radiothérapie et la chimiothérapie. Le pronostic reste controversé. Des études sur des séries plus larges sont nécessaires pour codifier la prise en charge.

## Conflits d’intérêts

Les auteurs ne déclarent aucun conflit d’intérêts.
